# Melanoma: From Melanocyte to Genetic Alterations and Clinical Options

**DOI:** 10.1155/2013/635203

**Published:** 2013-12-12

**Authors:** Corine Bertolotto

**Affiliations:** ^1^INSERM, U1065 (Équipe 1), C3M, 06204 Nice, France; ^2^University of Nice Sophia-Antipolis, UFR Médecine, 06204 Nice, France

## Abstract

Metastatic melanoma remained for decades without any effective treatment and was thus considered as a paradigm of cancer resistance. Recent progress with understanding of the molecular mechanisms underlying melanoma initiation and progression revealed that melanomas are genetically and phenotypically heterogeneous tumors. This recent progress has allowed for the development of treatment able to improve for the first time the overall disease-free survival of metastatic melanoma patients. However, clinical responses are still either too transient or limited to restricted patient subsets. The complete cure of metastatic melanoma therefore remains a challenge in the clinic. This review aims to present the recent knowledge and discoveries of the molecular mechanisms involved in melanoma pathogenesis and their exploitation into clinic that have recently facilitated bench to bedside advances.

## 1. The Melanocytes: From Photoprotection to Cancer

### 1.1. Melanocyte Development

**Figure 1:**
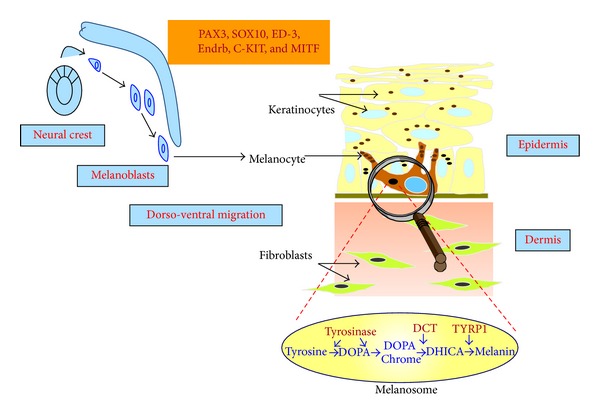
General overview of melanocyte physiology. Melanocytes derived from the neural crest in the form of undifferentiated and unpigmented precursors, the melanoblasts, migrate to their final destination, the epidermis, where they synthesize melanin in melanosomes. Pax3, Sox10, endothelin3 (ED-3) and its receptor (Endrb), c-Kit and Mitf play a critical role in the development of melanocytes. Melanin is then transferred to neighboring keratinocytes to ensure skin protection against the deleterious effect of ultraviolet radiation.

Melanoblasts undifferentiated and unpigmented precursors migrate from the neural crest to their final destination, the epidermis and hair follicles, where they differentiate and become mature melanocytes able to synthesize and transfer melanin pigment to neighbouring keratinocytes (Figure 1). Melanocytes are also found in the stria vascularis of the inner ear cochlea where they are involved in the production of endolymph along with ion exchange essential for hearing. Melanocytes are also located in the iris and in the choroid where pigments are involved in the formation, behind the retina, of the darkroom, which is necessary for the vision. This review will focus on cutaneous melanocytes only.

During embryogenesis, the survival and migration of melanocytes rely on signaling pathways such as Wingless signaling (Wnt)/*β*-catenin, the endothelin B receptor and its ligand endothelin-3, the receptor tyrosine kinase KIT and its ligand KIT-ligand/SCF (stem cell factor), NOTCH [1, 2], and transcription factors activity such as paired box gene 3 (PAX3), SRY (sex-determining region Y)-box10 (SOX10), hairy/enhancer of split (HES1), and microphthalmia-associated transcription factor (MITF) [3–7]. In humans, mutations in the genes encoding KIT, PAX3, SOX10, and MITF are the cause of various diseases, such as Piebaldism, Waardenburg, or Tietz syndromes, which are characterized by patchy depigmentation. Patients suffering from the Waardenburg or Tietz syndromes are also characterized by profound deafness [8, 9].

A pool of melanocytes with stemness properties (MSC) remains in the lower permanent portion (bulge) of the hair follicle, where these MSC can segregate during the hair cycle into mature melanocytes that color the new growing hair and into melanocyte-stem cells to maintain the pool of undifferentiated melanocytes. Maintenance of MSC also depends on the above signaling pathway and transcription factors [1]. Mouse models revealed that MSC are characterized by a low-level expression of Mitf, cKit, Sox10, Lef1, and Ednrb, which control melanocyte proliferation, an observation consistent with the quiescence status of the MSC. On the other hand, Pax3 and Dct expression were found upregulated [10]. Although the mechanisms which control this program remain to be fully elucidated, an explanation could be elevated expression of Wnt signaling inhibitors, such as Dkk3, Sfrp1, and Dab2, in the microenvironment [1, 11].

### 1.2. Physiological Role of Melanocytes

The main physiological function of skin melanocytes is to produce melanin pigments. The pigments are synthesized within specialized organelles called melanosomes through an enzymatic cascade involving tyrosinase, tyrosinase-related protein-1 (TYRP1), and tyrosinase-related protein 2/dopachrome tautomerase (DCT). Two types of pigments are produced, the brown/black pigment eumelanin which displays photoprotective features and the orange/yellow pigment pheomelanin endowed with poor photoprotective properties (Figure 1). Pigmentation is a heritable trait, being regulated by genetic factors, but the amount, type, and distribution of melanins in the skin, hair, and eyes can also be influenced by environmental and endocrine factors. Pigments have an extremely important role in our organism because they provide an efficient protection against the harmful effect of ultraviolet radiation (UVR). By absorbing and scattering UVR, melanin reduces the UVR-induced cellular DNA damage and genomic instability. This process is even more important upon tanning purposes and it responds to damage from UVR by producing more pigments. The central transducer of this response is p53 activation in keratinocytes, which, upon UVR, stimulates the transcription of proopiomelanocortin (POMC), the precursor of hormones such as *α*-melanocyte stimulating hormone (*α*MSH) or adrenocorticotropic hormone (ACTH), endowed with pro-pigmenting activities [12]. *α*MSH through activation of its melanocortin-1 receptor (MC1R) activates the cAMP/protein kinase A (PKA)/CREB signaling pathway and enhances the level of the transcription factor MITF [13, 14]. MITF is critically required for pigment production and a reduction in its activity is coupled to reduce melanin synthesis and pigmentation [15]. MITF also regulates expression of genes involved in melanosome biogenesis [16, 17], melanin synthesis [18, 19], and melanosome trafficking [20, 21]. Therefore, MITF coordinates an integrated cellular response for pigment production and function.

In addition to increased pigmentation, an increased number of melanocytes is found in UVR-exposed skin [22–24], a process that likely contributes to enhance photoprotection of the skin, through increased ability to provide skin with pigment. The proliferative ability of melanocytes mainly depends on keratinocyte-released factors such as *α*MSH, endothelin, granulocyte-macrophage colony-stimulating factor (GM-CSF), steel factor, leukemia inhibitory factor (LIF), basic fibroblast growth factor (bFGF), and hepatocyte growth factor (HGF) [25], which secretion may be stimulated by UVR. The growth factors, hormones, and ligands bind and activate their respective receptors that are connected to the MAPK/ERK and PI3K/AKT signaling cascades. Our lab provided the first demonstration of BRAF expression and its implication in melanocyte function by showing that BRAF activation via the *α*MSH/cAMP cascade led to an increase of proliferation upon the MEK/ERK signaling pathway activation [26]. This report was the beginning of what is now the biggest focus in the melanocyte field, the role of BRAF in melanoma disease. Activation of the ERK and PI3K signaling pathways are also associated with a survival program. Indeed, considering the key role of melanocytes in protecting our body against the noxious effect of UVR, melanocytes have developed potent antiapoptotic mechanisms involved in their resistance to the UVR-induced DNA damage and cell death. In this context, MITF whose expression is increased upon UVR controls the expression of antiapoptotic genes such as BCL2 [27], BCL2A1 [28], and ML-IAP [29] and several genes involved in DNA repair [30].

Exposure of human skinto sunlight has positive health effects in its ability to boost the body's vitamin D supply, which is essential for overall body health. The avoidance of all direct sun exposure increases the risk of vitamin D deficiency, which can have serious consequences, among which increased risks of deadly cancers [31, 32]. However, frequent and intense exposure to UV radiation sunlight, especially in childhood, is the major environmental risk factor for melanoma development.

These observations illustrate how important is the delicate balance between differentiation and proliferation/survival of melanocytes.

### 1.3. Melanoma Disease

Cutaneous metastatic melanoma (CMM) deriving from melanocyte transformation in 75% of the cases and from preexisting nevi in 25% of the cases is the most deadly form of skin cancer. Melanoma accounts for less than 5% of skin cancers but is responsible for 80% of skin cancer related deaths. Melanoma incidence in Caucasian population has increased dramatically worldwide during the past several decades. Melanoma is a cancer with a relatively good prognosis when diagnosed early at a cutaneous localized stage. Patients with stage 0/I thin lesions can usually be cured with surgical excision. Their 5-year survival rate ranges from 90% to 100%. However, the prognosis worsens the deeper the lesion extends beneath the skin, because of melanoma's propensity to invade and to metastasize. Individuals with thick melanomas have an increased risk to develop lymph node and visceral metastases. Metastatic melanoma cannot be completely removed by surgery and metastatic cells display extreme resistance to all types of treatment. Patients with metastatic melanomas have a median survival rate that typically ranges from six to ten months.

Melanomagenesis is a complex phenomenon in which environmental, genetic, and host factors play a role. Data from the clinic, epidemiology and more recently from genetic reveal that melanomas are heterogeneous tumors, harboring various genetic alterations, developing at different body sites and on sun-exposed and non sun-exposed regions, suggesting that melanoma arises from divergent causal pathways. Curtin et al. proposed a molecular classification based on the sites where the melanoma occurs, the genetic alterations and the sun exposure history [33]. Mutations in *BRAF* were significantly more common in melanomas located in areas without chronic sun-induced damage. Melanomas arising in chronically sun-damaged skin, mucosal surfaces, and acral skin were characterized by wild-type *BRAF* and wild-type *NRAS* but exhibited alterations in *KIT*.

#### 1.3.1. The Constitutional Risk Factors

We are not all equal in front of the sun. Major risk factors include skin phototype and skin reaction to sun exposure according to the phototype, a high number of nevi/dysplasic nevi and a personal and familial history of melanoma.

Subjects with red/blond hair, blue eyes, fair skin, and developing sunburns have higher melanoma risk than subjects with brown hair/eyes and skin that tan easily. The former produce little or no eumelanin and are therefore much less protected from the noxious effects of UV radiation. Subjects with >50 nevi or having at least five atypical moles exhibit fivefold higher risk of developing melanoma. The presence of large congenital nevi is also a melanoma risk factor [34]. Finally, approximately 10% of melanomas occur in a familial context, defined by at least two melanomas within two or more members of the same family. Rare deleterious germinal mutations in the cell cycle regulators *CDKN2A *and cyclin dependent-kinase 4 *(CDK4)* have been shown to confer a high cutaneous malignant melanoma risk [35, 36]. Additionally, the frequent allelic germinal variants (MC1R, ASIP, MTAP, MATP, and Casp8) have been identified as low-risk susceptibility genes or as modifiers of high-risk genes [37, 38]. Recently, a germinal mutation in the master gene of melanocyte homeostasis, microphthalmia-associated transcription factor (MITF), has been identified and shown to increase the risk of developing melanoma [39–42]. Finally, subjects who have had a melanoma have an approximately 9-fold increased risk of developing subsequent melanoma compared with the general population [43].

#### 1.3.2. Acquired Risk Factors

Epidemiologic studies revealed that sun exposure is the major known environmental factor associated with development of melanoma. According to the world health organization (WHO), changes in sun exposure habits and attitudes, that often resulted in excessive UV exposure, were recorded in the recent decades and is the main cause of the increase in skin cancer number.

Excessive sunlight exposure, associated with sunburn especially in childhood and early adolescence years, starts the transformation of benign melanocytes into a malignant phenotype. Results from Noonan et al., who used a mouse model expressing HGF derived by the metallothionein promoter, constitute proof-of-concept [44]. A single UV dose delivered at the neonatal stage was sufficient to induce melanoma in HGF/SF-transgenic mice after a relatively short latent period and with high cumulative incidence. Noteworthy, the dose roughly administrated to mice in this study corresponds to a sunburning dose of natural sunlight at midlatitudes in midsummer. No melanoma was detected when UVR was delivered at the adult stage.

Furthermore, one person's risk of developing melanoma doubles if they have more than five sunburns at any age. The majority of melanomas are located on intermittently exposed body sites such as the trunk in men and lower limbs in women [45].

Sun's ultraviolet rays are divided into wavelength ranges identified as UVA (315 to 400 nm), UVB (280 to 315 nm), and UVC (100 to 280 nm). UVC is filtered by the stratospheric ozone layer, and, in theory, it does not reach earth's surface. Of the UV solar radiation that does reach the earth, UVA (95%) and UVB (5%) promote deleterious effects on proteins and nucleic acids. UVB is thought to be more carcinogenic than UVA by inducing the formation of cyclobutane pyrimidine dimers (CPD) and 6-pyrimidine 4-pyrimidone photoproducts [46], while UVA mainly produces oxidative stress [47]. The frequency of CPD generation is three times higher and they are less efficiently repaired than 6-4 photoproducts. The UVB-induced lesions generate typical genetic mutations, C to T and CC to TT transitions, called the “UVB signature mutations.” Both UVA and UVB can also trigger DNA damage through oxidative stress and indirectly damage DNA and cause genetic alterations. Experiments using animal models showed that UVB, but not UVA, was melanomagenic [22, 48].

Therefore, UVR may contribute to melanoma development through combined genotoxic and mitogenic effects in melanocytes. DNA repair-deficient xeroderma pigmentosum A (Xpa) mice display higher melanocyte proliferation induction by UVB exposure [23], and individuals with XP mutations display a greatly elevated incidence of skin cancers including melanoma [49], thereby indicating that DNA-repair genes play an active role in melanoma initiation. DNA repair genes are also involved in melanoma progression by conferring a sort of metastatic genome stabilization during the metastatic process [50]. The molecular mechanism by which these genes are controlled are poorly elucidated yet functional genomic experiments unveiled that the master gene of melanocyte homeostasis MITF controls the transcription of several genes involved in DNA replication and repair [30].

Together, these observations point to the importance of effective DNA damage repair and genomic stability in melanocyte transformation and melanoma initiation.

The absence of UV “signature” mutations in genes relevant to melanoma such as BRAF, NRAS, CDKN2A, or even p53, which harbor typical UV signature in nonmelanoma skin cancers [51], questioned for years the role of UVR in melanoma disease. Numerous recent publications solved this mystery using “deep sequencing approaches” by showing frequent UV signature mutation in the genome of different melanoma types, thereby providing the first genomic evidence for a direct mutagenic role of UV light in melanoma pathogenesis.

In a first study the authors sequenced the coding exons of 518 kinases in several cancers including melanoma and genome sequencing of a malignant melanoma, and a lymphoblastoid cell line from the same person revealed that the dominant mutational signature was C>T transversion, reflecting DNA damage due to ultraviolet light exposure [52, 53]. Of the 33345 somatic base substitutions reported by Pleasance et al., almost 25000 were C>T changes and 360 were CC>TT changes. Additionally, a high frequency of G>T substitutions that might reflect transversion ensuing oxidative DNA damage has been detected [54]. A high frequency of C>T transversions was nevertheless also identified in non-sun-related cancers such as glioma, gastric, or colorectal cancers. More evidence came from three other studies in which they compared the mutational status of melanomas from different body areas such as melanomas from the trunk versus sun-shielded acral, mucosal, and uveal melanomas [55–57]. The rate of point mutations was the lowest in primaries from non-ultraviolet-exposed hairless skin of the extremities (3 and 14 per megabase (Mb) of genome). The mutational rate was scored intermediate in those originating from hair-bearing skin of the trunk (5–55 per Mb) and highest in a patient with chronic sun exposure (111 per Mb) [55]. Moreover, these studies pointed out to mutations affecting the biological function of proteins, such as PREX2 (phosphatidylinositol-3,4,5-trisphosphate-dependent Rac exchange factor 2)-a PTEN-interacting protein and negative regulator of PTEN which is mutated in 14% [55], PPP6C encoding a serine/threonine phosphatase mutated in 9–12.4% and RAC1, encoding a GTPase of the RAS superfamily of small GTP-binding proteins mutated in 5–9.2% [56, 57] of sun-exposed melanoma cases. Noteworthy, PPP6C mutations were more frequent in melanomas that were mutated for both BRAF and NRAS, while RAC1 mutations were more frequent in melanomas that were wild-type for both BRAF and NRAS [56, 57]. Therefore, these studies firmly implicated UV irradiation in melanomas and provided potential new therapeutic options.

## 2. **Melanoma Disease: From Molecular Biology to Clinic**

Tumor growth is the result of genetic and/or epigenetic alterations in key genes (“driver” genes), controlling processes such as proliferation, apoptosis, senescence, and response to DNA damage. These changes lead to the synthesis of modified/activated (for oncogenes) or hypofunctional/absent (for tumor suppressor genes or DNA repair genes) proteins. Changes in the stoichiometry and normal biological behavior of these proteins within the cells will promote an acceleration of the tumor progression. At the initiation stage, the genetic alteration can be germline. As we know that the prognosis of a patient is closely linked to its early diagnosis; identification of cancer predisposing genes is crucial to identify and monitor at-risk patients. This chapter provides an overview of the key molecular proteins and associated pathways implicated in the acquisition of the malignant melanoma phenotype.

### 2.1. Melanoma Susceptibility Genes

Historically, high-risk germline mutations in cancer predisposing genes (oncogenes or tumor suppressor genes) were discovered through linkage analysis in large pedigrees showing Mendelian-like mode of inheritance. During the last five years, genome-wide association studies (GWAS) have identified common SNPs associated with a low risk of developing cancers, including melanoma [58]. However, for many cancers, as for other complex diseases and human traits, the known loci explain only relatively small fractions of the total genetic variance, and the identification of genetic variants with low allele frequency conferring a moderate-risk of cancer through these classical approaches is much more challenging. Therefore, mutation screening of candidate genes (or of the entire exome, now achievable thanks to the latest improvement in sequencing technologies) in carefully selected at-risk patients, coupled with appropriate tools to assess the pathogenicity of the genetic variants (*in silico* and *in vitro* assays), is an important complementary approach of choice to identify the missing heritability.

Recent studies have demonstrated that common diseases can be due to dysfunctional variants with a wide spectrum of allele frequencies, ranging from rare to common. This is the case for example in breast cancer, where high-risk mutations in *BRCA1*, *BRCA2*,* PTEN*,   and* TP53*, intermediate-risk variants in genes of the DNA repair pathways (*ATM, CHEK2, BRIP1, and PALB2*), and low-risk SNPs (in *FGFR2*, *TOX3*, *CASP8*, *MAP3K1*,  and* LSP1*) have been identified [59].

**Figure 2:**
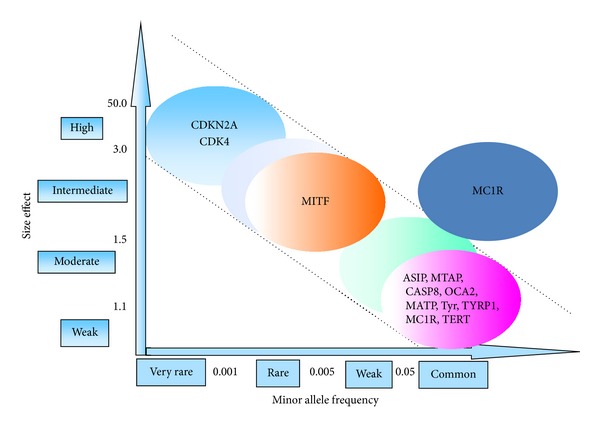
Melanoma susceptibility alleles (adapted from Manolio et al.).

Until very recently, these rare, sometimes private, moderate-risk susceptibility alleles have been identified through resequencing of candidate genes selected on the basis of biological plausibility. A candidate gene approach has been applied to identify the MITF E318K mutation. In this particular case, the fact that two studies reported the same recurrent *MITF* mutation in independent patient series/populations using an agnostic genome-wide approach (massive parallel exome sequencing approach plus genotyping in larger melanoma series, familial CMM, where multiple primaries often occur) reinforces the notion of the existence of variants with low minor allele frequency that could have substantial effects (Figure 2).

#### 2.1.1. Genes with High Penetrance

Rare alleles of CDKN2A and CDK4 genes have been identified in familial forms of melanoma among patients who have had melanoma. Mutations or deletions in these genes confer an elevated risk of developing melanoma. These genes are involved in cell cycle arrest and cellular senescence. Their importance is revealed by the fact that germline and somatic mutations in CDKN2A and CDK4 directly or in their associated signaling pathways are almost invariably found in melanomas.

*The CDKN2A Locus.* CDKN2A (Cyclin-Dependent Kinase Inhibitor 2A) is located at the 9p21 locus and encodes two tumor suppressor proteins p16^INK4a^ (Inhibitor of Kinase a) and p14^ARF^ (Alternative Reading Frame). p16^INK4A^ is transcribed from exons 1*α*, 2, and 3, with exon 3 encoding only four amino acids. p14^ARF^ is encoded by an alternative exon 1 (1*β*) spliced to *CDKN2A* exon 2 in an alternate reading frame (ARF). The p16^INK4a^ and p14^ARF^ transcripts are translated in different reading frames; thus the two proteins have no homology at the amino acid level [60]. In familial melanoma, which represents 8 to 12% of all melanomas [61], germline mutations in the CDKN2A gene are found in 20–40% of cases [62]. These mutations can affect p16^INK4a^, p14^ARF^, or both proteins. p16^INK4a^ interacts specifically with both CDK4 and CDK6 and blocks their association with D-type complexes [63]. Thus, the loss of function of p16^INK4a^ promotes CDK4 and CDK6 activation, resulting in hyperphosphorylation of pRB, and the activation of the transcription factor E2F1. E2F1 mediates the transcription of S phase promoting genes, thereby promoting cell proliferation.

Evidence of a role for p16^INK4a^ in human melanoma includes frequent somatic genetic and epigenetic alterations in human melanoma samples. Indeed, p16^INK4a^ gene is lost in 50% of melanoma cases, inactivated by point mutations in approximately 9% of tumors or inactivated by methylation of its promoter in about 10% of melanoma cases [64]. p16^INK4a^ loss of function promotes senescence bypass and melanocyte immortalization [65, 66]. Senescence is a program that represents a potent barrier against tumorigenesis by preventing the proliferation of cells at risk for neoplasic transformation. The reintroduction of a functional copy of p16^INK4a^ in melanoma cell lines induced a significant change in morphology associated with dendricity, a parameter of differentiation, and decreased cell growth [67].

The collaboration of oncogenic NRAS^Q61K^ and p16^INK4a^ loss of expression is sufficient to cause melanoma formation in mice supporting the critical role of p16^INK4a^ in melanoma aetiology [68].

Although p16^INK4a^ was thought to be the predominant tumor suppressor at 9p21, observations from melanoma-prone families in which exon 1*β* germline deletion or mutation in either the coding region or splice donor site of this exon have been reported supporting a p16^INK4a^-independent tumor suppressor role for p14^ARF^ [69–71]. A comprehensive analysis of the pattern of genetic and epigenetic alterations to the p16^INK4a^ and p14^ARF^ tumor suppressor loci in melanoma revealed that p14^ARF^ is frequently inactivated [72]. Supporting the role of p14^ARF^ in melanomagenesis, murine melanoma models demonstrated that specific inactivation of p14^ARF^ (p19ARF in mouse) enhanced melanoma development [73, 74]. Although p14^ARF^ is mainly known to function by preventing p53 degradation by the E3 ubiquitin-protein ligase MDM2 (Mouse Double Minute 2), in mouse context, melanomagenesis in ARF-depleted background is p53-independent [73, 74].

Consistently, activating mutations of BRAF and loss of functional p16^INK4a^ and p14^ARF^ were detected in the majority of melanomas [75].

*CDK4.* Germline mutations in the gene encoding cyclin dependent kinase 4 (CDK4A) have been identified in a very small percentage of familial melanoma [36, 76]. The mutation of arginine at position 24 into cysteine (CDK4^R24C^) or histidine (CDK4^R24C^) renders the protein insensitive to regulation by p16^INK4a^ but preserves interaction between CDK4 and cyclin D1 leading to constitutive activation of the complex and aberrant proliferation, through retinoblastoma protein inactivation and E2F activation. CDK4^R24C^ facilitates tumorigenesis of melanocytes transplanted into nude mice and causes escape from cellular senescence [77]. Mouse models show that the CDK4^R24C^ form enhances melanoma penetrance in cooperation with the oncogenic HRas^G12V^ [78], which is found in the subset of atypical spitzoid melanomas or after specific carcinogenic treatments [79], thereby supporting the notion that CDK4 is important for melanoma development.

#### 2.1.2. Intermediate/Low Penetrance Genes

Minor familial melanoma loci have been more commonly found in the general population with a lower penetrance of their germline mutation than CDKN2A and CDK4.

*MITF.* MITF belongs to the MYC supergene family of basic helix loop helix transcription factors. MITF is critical for melanocyte cell-fate determination during commitment from pluripotent neural crest stem cells and is also required for postnatal melanocyte functioning [80]. While MITF has only been considered for many years as the master regulator of melanocyte differentiation, more recent data have implicated MITF in the control of proliferation, survival, and the pathogeny of melanoma [81]. Genomic amplification of MITF is found in 10% of primary and 20% of metastatic melanomas and correlates with decreased 5-year overall patient survival, yet it does not translate into a strong increased expression at the protein level [82, 83]. The paradoxal function of MITF could be explained by a variation in its level of expression, its different cofactors, and its posttranslational modifications [84]. It has been proposed that MITF acts as a rheostat in the melanocyte lineage. In this model, a transient decrease in MITF expression is associated with a melanoma-initiating cell phenotype [85–87], whereas moderate MITF level is linked with proliferation [84, 88] and high MITF level with differentiation [14, 18, 19]. Moreover, a sustain inhibition of MITF expression is associated with a senescence phenotype [30, 89–91].

Posttranslational modifications of MITF also contributes to MITF activity. Therefore, MITF can be phosphorylated and degradated via the ubiquitin-proteasome pathway in response to activation of the ERK pathway [92, 93]. By sequencing the entire coding sequence of *MITF* in a highly selected set of patients presenting either a strong family history of CMM or multiple primary melanomas, our group identified a germline missense substitution p.E318K (c.952G>A, NM_000248.3), occurring at a significantly higher frequency in the at-risk patients than in the control population and conferring a 5-fold increased melanoma risk [39]. The mutation was also reported in sporadic and familial cases in Australian and English cohorts and associated with a 2-fold increased melanoma risk [42]. Subsequently, the *MITF *E318K variant was found in a group of Italian melanoma patients [40] and in another Australian study [41] with similar allele frequency. All these studies show that the *MITF E318K* variant is enriched in those with multiple primary melanomas.

It was shown that the nonsynonymous c.952G>A substitution that changes the glutamate 318 into lysine replaces the SUMOylation consensus binding site IKQE in the C-terminal part of MITF by the IKQK sequence and reduces MITF sumoylation [39]. SUMOylation is an ubiquitination-like posttranslational modification triggering covalent SUMO attachment to target proteins [94] which deregulation has been involved in several human diseases.

Expression of MITF E318K enhances the migrative and invasive properties of melanoma cells and increases the ability to form colonies of immortalized melanocytes, hence indicating that MITF E318K displays protumoral properties [39].

According to its level and the type of posttranslational modification, the repertoire of MITF target genes differs. MITF has been reported to control expression of genes involved in cell proliferation (CDK2) [95], in cytoskeleton remodeling and migration [84], and in cell survival (BCL2, BCL2A1, ML-IAP, MET, APE1, and HIF1a) providing antioxidant defense and elevating the antiapoptotic features of melanocyte cells [27, 28, 96–98]. Consistently, MITF activity correlates directly with resistance to UV-induced apoptosis in melanocytes [99]. MITF is also the major transcriptional regulator of *TRPM1*, a transient receptor potential cation channel, which expression has been correlated with a higher metastatic risk in skin melanoma [100]. Recently, MITF was reported to regulate the expression of PGC1*α* [101, 102], a regulator of mitochondrial biogenesis and respiration, gluconeogenesis as well as many other metabolic processes [103]. More broadly, MITF occupies a prominent position in the melanocyte lineage, working as a molecular *hub* that determines the melanocyte behavior.

*MC1R.* The melanocortin 1 receptor (MC1R) is a seven-pass transmembrane G-protein coupled receptors, expressed on the cell surface of epidermal melanocytes. Upon stimulation by the proopiomelanocortin-derived peptides *α*-melanocyte-stimulating hormone (*α*-MSH) and adrenocorticotropic hormone, MC1R activates adenylate cyclase, the cAMP/PKA/CREB cascade, and the pigmentation phenotype. Loss-of-function for *MC1R* is responsible of the so-called red hair color phenotype in individuals of northern European ancestry. Additionally, genetic variation at *MC1R locus* is also an important risk factor for melanoma [104, 105]. Therefore, carriers of MC1R variants that correspond to the red hair, fair skin phenotype (V60L, R151C, R160W, and D294H) have a 2–4-fold increased risk of developing melanoma [106–108]. Moreover, the presence of an MC1R variant in addition to a CDKN2A mutation significantly increases the melanoma penetrance, decreasing the age at onset compared with individuals carrying a CDKN2A mutation alone [109, 110]. Finally, patients with *MC1R* variants had a 5- to 15-fold increased risk of *BRAF*-mutant melanomas regardless of signs of chronic solar damage [111, 112].

*Other Low-Risk Alleles.* Recent studies have focused on different variants responsible for differences in pigmentation of hair, eyes, and skin but also skin sensitivity to UV. It has been shown that variants of pigmentation loci such as in ASIP, an MC1R receptor antagonist competing with the *α*-MSH/MC1R cascade and thus inhibiting the constitutive pigmentation, the melanogenic enzymes TYR and TYRP1 [113] or MATP/SLC45A2 (membrane-associated transporter protein/solute carrier family 45 member 2) involved in intracellular processing and trafficking of melanosomal proteins [114] are significantly associated with increased melanoma risk [113]. In addition, genes not involved in pigmentation were associated with an elevated risk of developing melanoma, including germline mutations in MTAP (methylthioadenosine phosphorylase), an enzyme playing a major role in polyamine metabolism [115] and PLA2G6 encoding a phospholipase A2 group VI [115]. The *TERT* locus (at 5p15.33) was found associated with melanoma risk. Recently, a melanoma-segregating germline mutation in the promoter of the telomerase reverse transcriptase (*TERT*) gene has been shown to create a new binding motif for Ets/TCF transcription factors such as *ELK1* and *ELK4*, near the transcription start. In reporter gene assays, this variant caused up to 2-fold increase in transcription [116].

### 2.2. Acquired Genetic and Epigenetic Components

Genes can also be targeted for mutations or deletions not in the germline but as acquired events in individuals with sporadic melanomas. Melanoma is a heterogeneous tumor and several molecular events revealed by genomic, proteomics, and candidate gene approaches have been identified and associated with its development. In addition to the commonly mutated genes BRAF, NRAS, PTEN, TP53, and p16, new candidate genes have been identified. GRIN2A, which encodes a subunit of the glutamate receptor [117], ERBB4, a growth factor transmembrane receptor [118], and the metalloprotease MMP8 [119] are mutated in 30%, 19%, and 7% of melanoma cases, respectively. More recently, deep-sequencing approaches of melanoma samples of different melanoma types highlighted new melanoma driver genes such as PREX2, PPP6C, and RAC1 [55, 56]. Other mutations were also reported such as in SNX31 that encodes the protein sorting nexin 31 likely acting as a Ras effector protein [120], in TACC1 that stimulates the Ras and PI3K pathways and promotes transformation *in vivo* [121], and in STK19, a kinase with yet unknown function. Functional studies are necessary to clearly identify the role of some of these recent mutations in melanoma pathogeny.

Rather than establishing a catalog of all the molecular changes reported so far in melanoma tumors, this review will be restricted to the main players of melanomagenesis. In addition to environmental factors, cells must acquire successive genetic lesions prior to forming tumors. These alterations are discussed using a conductive wire based on the known biological role of these players in melanoma initiation and progression.

**Figure 3:**
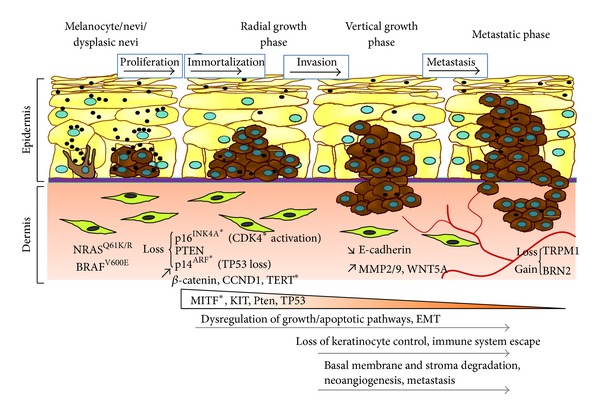
Hypothetical model of Melanoma development. In 25% of cases, melanoma derives from a pre-existing nevus through a multistep process regulated by a key set of genes. Cells must acquire successive genetic lesions prior to forming tumors and metastases. Asterisks indicate genes mutated in the germline.

In aggregates, a high frequency of activating *BRAF* mutations (80%) was identified in nevi, indicating that activating *BRAF* mutations occurs early during melanoma progression [122], an idea supported by recent findings showing that the *BRAF* ^V600E^ mutation was present in the majority of, if not all of, melanocytes in the *BRAF* ^V600E^ nevi examined [123]. Activating *BRAF* mutations triggered an initial burst of cell proliferation followed by induction of senescence. The primary mediator of *senescence* in *nevi* appeared to be p16^INK4a^ blocking cyclin D1/CDK4 complexes and preventing cell proliferation. KIT also mediated cell cycle stimulating activities yet its effect seemed to be restricted to a subset of melanomas. PTEN loss triggered activation of the PI3K/AKT pathway and escaped of BRAF^V600E^-mediated senescence. Therefore, PTEN loss could terminate the bypass of senescence initiated by p16^INK4a^ loss, and via PI3K/AKT pathway could favor melanoma progression. Deregulation of the PI3K/AKT pathway mostly occurred as a late-stage event in melanoma, implying that it operated in malignant progression more than in melanoma initiation. This notion was supported by a study in which the functional impact of the RAS/BRAF and PI3K/AKT signaling pathways was investigated using reconstructed skin [124]. Expression of the catalytic subunit of PI3K produced invasive melanocytic neoplasia while only mild junctional hyperplasia was seen upon BRAF^V600E^ expression. RAC1, one of the key target/effector of PI3K, regulates cell motility. The metastasis process is also associated with acquisition of mesenchymal properties, a process assimilated to an EMT program that is favored by the hypoxic environment of the skin [125] (Figure 3).

#### 2.2.1. The RAS/RAF/MEK/ERK Cascade

Ras family consists of three isoforms HRAS, NRAS, and KRAS each encoding a membrane-localized small GTPase. The members are composed of a catalytic domain that mediates the guanine nucleotide binding and hydrolysis and of an hypervariable region containing the membrane targeting domain required for its activation. They function as a molecular switch linking receptor and nonreceptor tyrosine kinase activation to downstream cytoplasmic or nuclear events. The family of serine/threonine kinases RAF has three isoforms ARAF, BRAF, and CRAF (RAF-1) activated by the small GTPases RAS.

Oncogenic RAS or BRAF alone appears relatively poor in inducing melanoma transformation unless they are combined with other genetic alterations. In human, 81% of congenital melanocytic nevi harbor RAS^Q61K/R^ mutations and 82% of acquired nevi harbor BRAF^V600E^ mutation [126]. Forced expression of oncogenic NRAS or BRAF in normal melanocytes triggers a senescence phenotype [127, 128], a notion that supports the idea that nevi have a growth arrest via oncogene-induced senescence. Nevi can remain arrested in growth for decades. Mouse models expressing NRAS^Q61K^ [68] or BRAF^V600E^ [129, 130] develop benign melanocytic lesions, characteristic of nevi, that rarely progress to melanoma, strengthening the previous notion. However, melanoma formation in mouse model is greatly accelerated in the absence of p16^INK4a^ [68], Pten [129], or *β*-catenin [131]. In human also, it is thought that several genetic/epigenetic alterations and/or additional environmental stimuli are needed to drive melanoma development [132].

Almost 80% of melanomas have either BRAF or NRAS mutations [133]. Most common melanoma mutations are in NRAS (15–30% of the cases), among which the most frequent are substitutions of glutamine at position 61 by a lysine or an arginine (Q61K, Q61R) [134]. However, HRAS mutation can also be found in the subset of sptizoïd melanomas [135]. In 2002, oncogenic mutations in the serine/threonine kinase BRAF was identified in nearly 70% of cutaneous melanomas [136], a percentage closer to 50% when considering a higher number of melanoma specimens [33, 137]. The T1796 → A transition leading to the V600E change at exon 15 represents nearly 90% of the cases. This mutation creates a constitutively active status for BRAF, independent of a previous activation by RAS oncogene and extracellular stimulus. BRAF mutations are associated with high level of UVR in early life compared to patients with NRAS mutation who have high total exposure spread throughout life. Patients with BRAF mutations are younger and have greater number of nevi. NRAS and BRAF mutations are mutually exclusive; yet very rare exceptions were reported [138].

Oncogenic BRAF transforms immortalized melanocytes [139] and it stimulates proliferation of melanoma cells. Similarly, introduction of MEK into murine immortalized melanocytes leads to tumorigenesis in nude mice [140]. Conversely, inhibition of Ras [141], BRAF [142–144], or MEK blocks ERK activity and inhibits the growth of melanoma cells both *in vitro* and *in vivo*.

Recently, NRAS/BRAF activation was shown to mediate an epithelial-to-mesenchymal transition (EMT) switch in late-stage melanoma that relies on TWIST1; ZEB1, and E-cadherin loss and results in enhanced invasion. This EMT program constitutes an independent factor of poor prognosis in melanoma patients [145].

This NRAS/BRAF signaling pathway has attracted considerable attention as a target for anticancer therapy because of its high frequency of mutations and its important role in melanoma disease. The novel cancer therapeutic approaches based on the inhibition of some members of this cascade will be developed and discussed later in Section 3.

#### 2.2.2. CCND1

The CCND1 gene encodes cyclin D1, an activating subunit of the CDK4 and CDK6 kinases which control the G0/G1 cell cycle progression. Cyclin D1 is a protooncogene playing an important role in cancer, including melanoma. Gene amplification of chromosome 11p13 containing the CCND1 locus and enhancing cyclin D1 expression has been observed in melanoma cell and particularly in acral (44,4%), lentigo malignant (10,5%), and sinonasal melanomas (62,5%) [146, 147]. Cyclin D1 overexpression might increase resistance to BRAF inhibitors [148], although this observation was not confirmed in further studies [149].

Cyclin D1 silencing triggers a G1/S cell cycle arrest *in vitro* [150] and leads to inhibition of melanoma growth *in vivo* [147].

#### 2.2.3. KIT

KIT is a type III transmembrane receptor tyrosine kinase. Binding of its ligand, stem cell factor (SCF), results in receptor dimerization, autophosphorylation, and activation of several signaling pathways, thereby, mediating cancer cell growth, proliferation, invasion, metastasis, and inhibition of apoptosis. The SCF/KIT pair plays a key role in melanocyte specification during embryogenesis and melanocytes differentiation and proliferation [151–153]. KIT is expressed in more than one-half of early-stage malignant melanomas [154]. However, loss of KIT expression has been observed with progression of disease from superficial and invasive to metastatic stages, suggesting that KIT possesses tumor suppressive functions [155]. Loss of KIT expression is thought to be mediated through AP-2*α* [156]. The AP2 transcription factor family is a set of retinoic acid inducible genes regulated along the development and composed of four related factors, AP2*α*, AP2*β*, AP2*γ*, and AP2*δ*. AP2 factors orchestrate a variety of cell processes including apoptosis, cell growth, and tissue differentiation during embryogenesis. AP2*α* has been shown to function as a tumor suppressor by regulating the transcription and expression of p53 [157]. A dominant-negative AP2*α* mutant enhances invasive and tumorigenic properties of melanoma cells [156]. KIT is activated by mutation in only 2–6% of cutaneous melanomas but aberrations in KIT (mutation, amplification) have been reported in melanomas of acral (36%), mucosal (39%), and chronic sun-damaged/lentiginous (28%) types [158–161]. It is interesting to note that in the majority of melanomas with mutations in BRAF or NRAS, the expression of c-KIT seems reduced to allow tumor progression [162]. However, an overexpression of KIT and CDK4 has been identified in a subgroup of cutaneous malignant melanomas and would be a mechanism of potential oncogenic transformation of nonmutated BRAF or NRAS melanomas [163].

#### 2.2.4. The Tumor Suppressor Gene TP53

The TP53 gene encodes the transcription factor p53 activated in response to various stresses including DNA damage, hypoxia, or expression of aberrant oncogene. p53 regulates positively or negatively many genes involved in cell cycle regulation (CDKN1A), induction of autophagy, senescence, and apoptosis (NOXA, PUMA, and BAX), as well as genes involved in the DNA repair or cellular metabolism [164].

The importance of p53 in maintaining cellular integrity is underscored by the fact the TP53 knockout mice spontaneously develop tumors [165]. Mutations or deletions of TP53 are found in approximately 50% of human cancers; yet in melanoma the ratio is lower. 1–5% primary melanomas and 11–25% metastatic melanoma harbor mutated p53 [166]. Most of the melanoma cases harboring p53 mutation (19% in the discovery set) were without concurrent mutation in CDKN2A locus [56]. Why frequency of p53 mutation is lowest in melanomas compared to other cancers remains to be solved. In human melanoma cells, deregulation in upstream regulators or downstream effectors indicate that the p53 signaling cascade is nevertheless compromised. Moreover, compelling evidence supports a role for p53 in melanomagenesis. Mouse and zebrafish models harboring oncogenic BRaf or Ras develop benign melanocytic hyperplasias that resemble nevi while melanomas occur in a p53-deficient background, thereby indicating that p53 restrains tumor progression [167–170]. Therefore, melanoma cells show a decreased expression level in the proapoptotic p53 effector APAF1, following methylation of its promoter [171, 172]. Additionally, MDM2, the E3 ubiquitin ligase responsible for p53 ubiquitylation and degradation via the proteasome, is frequently overexpressed in melanoma cell lines [173]. Recently, MDM4 over MDM2 has been shown to be overexpressed in cells freshly isolated from primary and metastatic melanomas compared to cultured melanoma cell lines, the later being likely selected for MDM2 overexpression [174]. These observations could explain why nutlin-3, an inhibitor of the MDM2-p53 interaction, provides poor clinical benefit. Strategies to impair the MDM4-p53 interaction are currently under development. Stapled peptides that bind with high affinity to MDM4 already provide promising antimelanoma effect both *in vitro* and in animal model experiments [174].

p53 negatively regulates hypoxia-inducible factor 1*α* (HIF-1*α*), a factor that facilitates melanoma invasion and development of more aggressive tumors [175, 176].

p53 also has a role in regulating both EMT and EMT-associated stem cell properties. p53 suppresses EMT by repressing expression of ZEB1, ZEB2, and SNAIL, through regulation of microRNA including members of the miR-200 family and miR34 [177, 178].

#### 2.2.5. The PI3K/AKT Signaling Pathway

The PI3K/AKT cascade can be activated by mitogenic stimuli or growth factors such as IGF1 or by RAS. PI3K catalyzes the phosphorylation of phosphatidylinositol (PI) into phosphatidylinositol-3 phosphate (PIP3), which recruits PDK1 and triggers the activation of the serine/threonine kinase AKT. Phosphatase and tensin homologue deleted on chromosome 10 (PTEN) is a phosphatidylinositol phosphate phosphatase and is frequently inactivated in human cancers. There are three forms of AKT, AKT1, AKT2 and AKT3. In melanoma cells, AKT3 is the form preferentially expressed. AKT3 activation is found in about 60% of sporadic melanomas subsequent to gene amplification (35% of the cases) or to inactivation of PTEN (40–60% of the cases), which negatively regulates the PI3K/AKT pathway [179–181]. Mutations of PI3K have been identified in only 5% of the cases [181]. Mutations in the PI3K signaling cascade were also discovered in MTOR, IRS4, PIK3R1, PIK3R4, PIK3R5, and NFKB1 [182].

Interestingly, while PTEN loss is found at high frequency in melanoma (37%) but not in nevi [181], AKT3 activity level increases with advancing melanoma stage [181]. These observations support the notion that alteration of the PI3K/AKT cascade is not an early event in melanoma progression.

NRAS and PTEN mutations were first reported to be mutually exclusive in melanoma, which is likely explained by their ability to share the same signaling pathway [183]. However, recent data indicate that PI3K pathway mutations cooccurred with 9% of NRAS mutant tumors [182]. Cooccurrence of BRAF and PTEN mutations was reported in 17% of melanomas [182].

Inhibition of AKT3 promotes an inhibition of tumor growth *in vivo* and is associated with an increase in apoptosis *in vitro* [181]. Reintroduction of PTEN reduces growth of melanoma xenografts [180].

More broadly, the PI3K/AKT signaling pathway contributes to the phenotypic plasticity of cancer cells by controlling EMT [184]. This pathway is also embroiled in invasion by regulating expression and activity of factors, such as RAC1, involved in cell motility and in degradation of basal laminae components, such as the metalloproteases MMP-9 [185], allowing melanoma cells to invade the underlying dermis [186].

#### 2.2.6. The Epithelial-Mesenchymal Transition

Epithelial-to-mesenchymal transition is the process in which epithelial cells lose their epithelial characteristics, gain mesenchymal features, and become motile. Cells that undergo EMT could also gain stem cell-like properties [187]. EMT orchestrates the lost of the cell-cell and cell-extracellular matrix (ECM) interactions and confers the ability to migrate, through induction of a new genetic program, specifically increased expression of SNAIL/SNAI1, SLUG/SNAI2, ZEB1, ZEB2/SIP1, TWIST proteins, and E47 and decreased expression of the adherence molecule E-cadherin [188–190].

EMT plays a crucial role during different stages of embryonic development [191] and can be reactivated in an adult organism under some circumstances such as tumorigenesis. EMT-promoting pathways are associated with different signaling molecules such as transforming growth factor beta (TGF-*β*), epidermal growth factor (EGF), fibroblast growth factor (FGF), hepatocyte growth factor (HGF), bone morphogenetic proteins (BMPs), and WNTs and Notch. Therefore, EMT programs emerge as important regulators of phenotypic plasticity in cancer cells.

EMT has been shown to play an important role in mediating the invasion and metastasis of epithelial tumors as well as melanoma cells [192]. In order to invade the underlying epidermis, melanoma cells will downregulate some molecules involved in cell-cell adherent junctions, such as E-cadherin, leading to their detachment from the basal laminae and to adjacent keratinocytes. Melanomas do not show a classical EMT and they adopt more mesenchymal features. E-cadherin expression may be repressed at the transcriptional level by Slug, Snail, or Twist [193–195]. In this regard, SLUG might facilitate the invasive properties of melanocytic cells [196]. In the context of melanoma, experimental regulation of E-cadherin expression controls cancer progression in some mouse models [197]. E-cadherin reintroduction decreases cell growth, survival, and invasion [198]. Interestingly, the level of E-cadherin correlates with MITF expression [199].

EMT is also regulated by the PI3K/AKT pathway through several routes. AKT enhances Mdm2-mediated ubiquitination and degradation of p53 [200] and p53 regulates the epithelial-mesenchymal transition [201]. Wnt signaling also regulates GSK-3*β* and thereby supports EMT by downstream effects on SNAIL and SLUG [202]. Additionally, AKT and a downstream effector RAC1 increase Snail expression [203, 204].

These observations support the role of the PI3K/AKT pathway in conferring melanoma cells invasive and stem cell-like features.

#### 2.2.7. TGF*β*

TGF-*β* signaling regulates melanoma tumorigenesis and metastasis [205] and is a major inducer of EMT [206]. The members of the SMAD family are activated by binding of TGF-*β* ligands to their cellular receptors, then they accumulate in the nucleus where they control the transcription of target genes. GLI2, a critical Sonic hedgehog mediator, has been identified as a direct transcriptional target of the TGF-*β*/SMAD pathway in melanoma cells [207]. High GLI2 expression is associated with a more aggressive phenotype characterized by loss of the cell-cell adhesion molecule E-cadherin, hallmark of cancer progression [208].

Increased expression and secretion of TGF-*β*2 and TGF-*β*3 seem to occur early in melanoma progression and to increase with tumor progression. A correlation between TGF-*β*2 expression and tumor thickness has been reported and TGF-*β* is associated with an invasive signature [209].

#### 2.2.8. The Wnt/*β*-Catenin Signaling Pathway

WNT are secreted glycoproteins involved in developmental processes and in the maintenance of cellular homeostasis, such as cell proliferation, cell polarity, stem cell self-renewal, and cell-fate determination [210]. Particularly, the WNT signaling cascade plays a crucial role in the neural crest induction, specification, and melanocyte differentiation.

The Wnt signaling might reflect the combination of at least three different signaling branches: the Wnt/*β*-catenin canonical branch, controls cell proliferation, and differentiation and the noncanonical branch involving the Wnt/Ca2+ and planar cell polarity (PCP) pathways is involved in cytoskeleton organization and cell motility.

Wnt1 and Wnt3a are considered as the canonical ligands and Wnt5a as an activator of the Wnt/Ca2+ pathway.

In the canonical pathway, binding of the Wnt proteins to their receptor, Frizzled (Fz), and coreceptor of the LRP family (only LRP5/6 are expressed in vertebrates) increases the pool of cytoplasmic *β*-catenin by preventing its phosphorylation and degradation through a complex including the tumor suppressor Adenomatous Polyposis Coli (APC), the scaffold protein Axin2, casein kinase 1 (CK1), and Glycogen Synthase Kinase 3 (GSK3). This leads to *β*-catenin translocation to the nucleus where it transactivates transcription factors of the TCF/LEF family and controls the transcription of target genes such as cyclin D1, c-MYC, MITF, and BRN2 and stimulates cell growth [7, 211–213]. TGF-*β* is known to activate the canonical Wnt pathway. This pathway is activated in more than 30% of melanomas, as illustrated by the presence of *β*-catenin in their nucleus [214] with approximately 3% harboring *β*-catenin mutations [215].

In mice, a form of *β*-catenin, constitutively localized at the nucleus, is not sufficient by itself to induce the formation of melanoma but causes melanocyte immortalization [216]. To do so, *β*-catenin seems to repress the promoter activity of p16^INK4a^, which controls the senescence program and prevents cellular immortalization. However, an active form of *β*-catenin increases the penetrance and incidence of melanoma in mice expressing oncogenic NRAS^Q61K^ or BRAF^V600E^ specifically in melanocytes [131, 216].

Additionally, expression of negative regulators of canonical Wnt signaling pathway such as Dickkopf-1, 2, 3 (Dkk-1, 2, 3) and Wnt inhibitory factor-1 (WIF-1) is strongly reduced or lost, both in melanoma cell lines and tumor samples [217, 218]. Forced expression of DKK1 or WIF-1 reduces melanoma cell growth and activates cell death [219, 220].

However, the role of the Wnt/*β* catenin pathway in melanomagenesis is complex and contradictory data have been reported. Metastatic progression is associated with the loss of nuclear *β*-catenin and the accumulation of *β*-catenin in cell nuclei of both primary tumors and metastases is a marker for good prognostic for patients [221].

Additionally, B16 melanoma cells expressing WNT3A implanted into mice exhibit decreased tumor size and decreased metastasis. Moreover, WNT3A upregulates genes involved in melanocyte differentiation, several of them are downregulated with melanoma progression [221]. Consistently, meta-analysis of melanoma gene expression revealed that the Wnt pathway is associated with high proliferation and low metastatic features [222]. Furthermore, BRAF signaling, activated in most of melanomas through mutation or autocrine/paracrine activation, inhibits Wnt/*β*-catenin signaling in human melanoma cells and *β*-catenin is required for PLX4720-induced apoptosis in melanoma cells [223].

Collectively, these discrepancies could be explained by the type of Wnt ligands and signaling cascades initiated by the level of nuclear *β*-catenin and the tumor stage.

Nevertheless, elevated expression of WNT5A is frequently associated with high grade melanomas [224, 225]. WNT5A forced expression in low metastatic melanoma increases their aggressive features [225].

#### 2.2.9. RAC1

The Rho family genes include more than twenty members and encode GTP hydrolases. The most extensively characterized members are RAC1 and RHOA [226]. These GTPases coordinate various cellular functions, including cell polarity, motility, vesicular trafficking, cell cycle, and transcriptomal dynamics [227, 228]. RAC1 interacts with p21-activated protein kinase 1 (PAK1) to regulate downstream events essential in tumorigenesis. Recently, a mutation in RAC1 has been discovered in melanoma and ranked as the third most frequent hot spot gain-of-function mutation occurring in melanoma after those in *BRAF* and *NRAS*. This RAC1-P29S substitution releases the conformational restraint conferred by the conserved proline, which induces an increased binding of the protein to downstream effectors and promotes melanocyte proliferation and migration [57, 229].

Additionally, aberrant activation of upstream regulators of RAC1, particularly in the DBL family of guanine nucleotide exchange factors (GEF) specific for RAC1 (e.g., TIAM1, PREX1-2, and ECT2), has been implicated in various cancers. Knock-out of PREX1, upregulated during melanomagenesis, reduces melanoma metastasis [230]. Recently, mutations in another member of the P-REX family, PREX2, have been reported; yet their role in melanoma disease remains to be clarified [55].

Rac1 inhibition, using pharmacological or genetic approaches, was reported to impair melanoma tumor growth and spreading to distant organs in the Tyr::NRas (Q61K) mouse model, suggesting a potential value for RAC1 as a therapeutic target, at least in some context [231].

## 3. Current and Emerging Approaches in Melanoma Treatment

As mentioned above, early stage melanoma localized to the skin can be cured by surgical excision. But most patients with unresectable stage III or stage IV disease require systemic treatment.

### 3.1. Conventional Chemotherapies

Conventional chemotherapy is based on the use of alkylating agents such as fotemustine (Muphoran), dacarbazine (Deticene), and temozolomide (Temodal) which trigger cytotoxic effects by blocking cell replication. However, these chemotherapy drugs promote only 10% of objective response with no improvement of overall survival [179]. Since the major breakthrough realized in 2011 with the FDA approval of vemurafenib (V600E mutated BRAF inhibition) for mutated BRAF^V600E^ melanomas, these drugs are limited to patients harboring non-BRAF^V600E^ mutated melanomas or for patients who developed resistance to previous treatments.

### 3.2. Personalized Therapy

The recent characterization of the molecular alterations in melanoma leads to the development of targeted therapies. These treatments are designed to target tumors according to their molecular diversity and activated intracellular signaling pathways.

#### 3.2.1. Targeting the BRAF/MEK/ERK Signaling Pathway

The discovery that BRAF is activated by mutation in a high percentage of melanoma specimens opened the door to the search for BRAF inhibitor. The first agent developed to target oncogenic BRAF was sorafenib (BAY 43-9006, Nexavar), a multikinase inhibitor that inhibited BRAF (wild-type or V600E) but also VEGFR, PDGFR, cKIT, and FLT3. This drug proved to be inefficient in the treatment of unresectable or metastatic melanoma [232]. Significant efforts have been spent to develop more specific and effective BRAF inhibitors leading to the discovery of vemurafenib (previously known as PLX-4032, currently marketed as Zelboraf), the first drug targeting mutated BRAF^V600E^ while having no/few effect on wild-type BRAF. In phases 1 and 2 clinical trials, vemurafenib showed an objective response rate >50% in patients suffering from melanoma. The results were confirmed in a phase 3 clinical trial (BRIM3), where unpreviously treated patients (*n* = 675) showed response rates of 48% for vemurafenib versus 5% for dacarbazine, a PFS of 5.3 months for vemurafenib versus 1.6 months for dacarbazine and at six months the overall survival was 84% in the vemurafenib group and 64% in the dacarbazine group [233]. With a median follow-up of 12.5 months for patients treated with vemurafenib and 9.5 months for those initially receiving dacarbazine, the PFS was significantly improved with vemurafenib (6.9 versus 1.6 months for dacarbazine), the overall survival was significantly prolonged with vemurafenib (13.6 versus 9.7 months) and the objective response rate was significantly higher with vemurafenib (57% versus 8.6%).

Vemurafenib received the FDA approval in 2011. However, a subset of BRAF^V600E^ patients were initially resistant to vemurafenib and most of the others developed secondary resistance. Nearly all tumors demonstrated reactivation of the MAP kinase pathway with elevation of ERK phosphorylation at the time of resistance. Different mechanisms involved in acquired and secondary resistance have been reported [234]. Moreover, a metabolic rewiring linked to oxidative phosphorylation and controls by the MITF/PGC1*α* axis has been involved in vemurafenib resistance.

Furthermore, vemurafenib showed adverse effects such as the development of cutaneous squamous cell carcinomas (SCCs) [235], through paradoxical activation of MAPK signaling (about 20–25% of the patients with advanced melanoma) [236].

Therefore, there is an urgent need to subvert an eventual drug resistance and/or further improve the clinical outcome of the patients.

In this context, other BRAF inhibitors are under development. Dabrafenib (marketed as Tafinlar) has received an FDA approval for unresectable or metastatic melanoma with BRAF mutations in 2013. Dabrafenib is in the same class as vemurafenib, working with a similar efficiency, but seems to be more efficient in melanoma brain metastasis [237]. In the pivotal phase 3 clinical trial (*n* = 250), dabrafenib significantly increased the response rate compared to dacarbazine (nearly 50% versus 6%) and PFS (median 5.1 months) compared with dacarbazine (median 2.7 months). Cutaneous side effects are also common with dabrafenib.

Therapeutic combinations are actually launched to replace single-agent BRAF inhibitors. Combination of a BRAF inhibitor and a MEK inhibitor exhibited reduced incidence of skin toxicity including the development of skin cancers, presumably because the MEK inhibitor blocks this paradoxical activation of the MAPK pathway.

Trametinib and MEK162 are potent, highly specific inhibitors of MEK1/MEK2, BRAF downstream kinases [238–240] giving responses in 20% of the melanomas harboring a BRAF mutation.

Trametinib (marketed as Mekinist) has also been FDA approved for unresectable or metastatic melanoma with BRAF V600E or V600K [239]. Its efficiency was demonstrated in the phase 3 Metric trial (*n* = 322) where PFS for trametinib was improved compared with either dacarbazine or paclitaxel (median 4.8 versus 1.5 months) as well as overall survival (81% versus 67%, resp., at 6 months).

#### 3.2.2. Targeting KIT

KIT is mutated in 15%–20% of patients with acral or mucosal melanomas and with melanoma occurring in areas of chronic skin damage. Imatinib mesylate, a kinase inhibitor targeting bcr-Abl, c-kit, platelet-derived growth factor receptor (PDGFR)-alpha and PDGFR-beta and inducing remarkable clinical responses in several cancers, was therefore used for melanoma. Although many activating c-KIT mutations have been reported, c-KIT mutant melanoma appears initially sensitive to imatinib [241]. It has been demonstrated *in vitro* that the tyrosine kinase inhibitor imatinib mesylate (Glivec) inhibits proliferation and induces apoptosis in melanoma cells with hyperactivation of c-KIT. These biological effects go through an increase in p27^KIP^ and inhibition of the ERK, PI3K/AKT, and STAT signaling pathways [163, 242]. However, Todd et al. have showed that the majority of patients treated with this inhibitor would eventually progress. Secondary mutations in c-KIT were reported to mediate this resistance. Cells with an A829P c-Kit mutation are resistant to imatinib but are still sensitive to the tyrosine kinase inhibitors nilotinib and dasatinib. Additionally, the T670I c-Kit mutation mediates resistance to imatinib, nilotinib, and dasatinib but remains sensitive to sunitinib [241].

Phase II studies using imatinib in unselected groups of patients with advanced melanoma demonstrated only minimal evidence of activity [243, 244]. However, phase II clinical trials conducted on patients with c-KIT mutations showed objective response rates in 33% of cases [245, 246]. Moreover, results from a phase II trial indicated that imatinib could be effective when tumors harbored KIT mutations, but not if KIT only was amplified [247].

### 3.3. Immunotherapy

It has been well documented that melanoma was an immunogenic tumor but metastatic melanoma cells have developed mechanisms to escape from immunosurveillance and to survive. The immune system involvement in protection against melanoma is supported by the increased melanoma incidence under immunosuppression conditions. Conversely, some melanomas which showed spontaneous regression and spontaneous infiltration of the tumor by T-lymphocytes seemed to be a factor of good prognosis [248]. These data led to the conclusion that immunological strategies could improve the prognosis of metastatic melanoma [249]. In 1998, the FDA approved the use of the immune molecule interleukin-2 (IL-2) to treat advanced melanoma. The immune molecule interferon alpha (IFN*α*) has also been used alone after surgery or in combination with other agents to treat advanced melanoma. However, most of clinical trials based on immune system activation did not translate into clinically meaningful objective response rate and any improvement in overall survival.

Therefore, development of novel therapeutic approaches, along with optimization of existing therapies, continues to hold a great promise in the field of melanoma therapy research. Promise for the use of immunotherapy in the treatment of melanoma has been highlighted recently.

#### 3.3.1. CTLA4 Blocking Antibody

In tumors, ectopic cytotoxic T lymphocyte-associated antigen 4 (CTLA-4) bounds to its ligands B7.1 and B7.2 on T-cells and caused inhibition of T-cells activity [250]. Following T-cell activation, CTLA-4 was recruited to the plasma membrane, where it functioned in an autoregulatory role, attenuating T-cell activation and proliferation, thereby restraining effective antitumor immunity. These observations suggested the potential value of an anti-CTLA4 approach in the treatment of metastatic melanoma. Ipilimumab (Yervoy), a novel antibody blocking CTLA-4, induced an overall survival benefit in two randomized phase III studies (*n* = 676 and *n* = 502, resp.) [251, 252] and received an FDA approval in 2011.

The median overall survival was 10–11.2 months among patients receiving ipilimumab plus gp100, or ipilimumab plus dacarbazine as compared with 6.4 months among patients receiving gp100 alone [251] and 9.1 months among those receiving dacarbazine [252].

The median overall survival with ipilimumab alone was 10.1 months [251]. Major drawbacks of this treatment were the low rate of objective response (10%), a minority of patients achieving long-term disease control, and the serious side effects.

#### 3.3.2. PD1 Blocking Antibody

The programmed death 1 (PD-1) receptor is an inhibitory receptor expressed at the surface of activated T cells. When PD-1 attaches to programmed death ligand-1 (PDL-1), expressed on cancer cells, the T cell's ability to target the tumor cell is inhibited.

Recently, monoclonal anti-PD-1 antibody lambrolizumab (MK-3475) was assessed in metastatic or unresectable melanomas (*n* = 135). Objective response rate was obtained in 38% of patients and the responses were sustainable in the majority of patients (median follow-up, 11 months among patients who had a response) [253].

Additionally, according to a phase 1 trial result including patients receiving a combination of nivolumab (BMS-936558), another PD-1 blocking antibody, and ipilimumab (*n* = 53) versus patients who received a sequenced treatment (*n* = 33), the combo provided deep, rapid, and long lasting tumor responses in patients with advanced melanoma. 53% of patients with a concurrent therapy demonstrated an objective response compared to the 20% objective-response rate of the group with sequenced treatment [254].

## 4. The Melanoma-Initiating Cells

Stem cells are undifferentiated cells characterized by the property of self-renewal. They can multiply into identical cells almost indefinitely or differentiate into specialized cells. Melanocyte stem cells (MSC) have been identified in the bulge of hair follicles [255]. They self-renew to maintain the pool of MSC and differentiate into functional melanocytes whose function is to color the growing hair. Incomplete MSC maintenance through SOX10, MITF, or Bcl2 loss triggers hair graying [255, 256]. A pool of MSC has also been identified from the dermis of glabrous skin by M. Herlyn's group; yet their function remains to be elucidated [257].

Tumor cells with stemness features have also been characterised in several cancers, including melanoma where differentiated and melanotic cells endowed with fast-growing properties, coexisting with cells growing slowly. These slow-growing cells are thought to mediate chemoresistance and relapses. Therefore it appears of paramount importance to determine their biological properties to develop efficient antimelanoma therapies. Thus, within a tumor, tumor cells proliferating rapidly but not individually competent to generate a new tumor-initiating cell can be distinguished from tumor cells proliferating very slowly but are able to generate a tumor. The existence of melanoma-initiating cells has only been recently proposed, with the description of a subpopulation of melanoma cells expressing markers such as CD20 [258], CD133 [259], CD24 [260], CD271 [261], or the ABC transporter (ATP-binding cassette) involved in drug efflux (ABCB5) [262], ALDH1A [263]. According to these studies, the initiating cells, which represent 1.6 to 20% of melanoma cells, were able to differentiate, self-renew, and establish clinically heterogeneous tumors in mice [258, 259, 262]. For example, the use of an antibody directed against ABCB5 prevented tumor formation or decreased tumor growth of a tumor already established [262].

Later on, several groups have validated the presence of melanoma-initiating cells identified as a minor subpopulation of cells with slow growing properties but with a high tumorigenic potential *in vivo* [85, 264]. These cells were required for tumor maintenance and to form a heterogeneous tumor when implanted into immunocompetent mice. Inhibition of this cell subpopulation either by forcing their differentiation [85] or promoting a metabolic rewiring [265] decreased tumor formation. Although the cellular heterogeneity found in the tumor might reflect the existence of different cell types, the studies also suggested the existence of a phenotypic transition allowing the same cells to acquire different phenotypes [87]. Indeed, JARID1B-negative cells could become positive [264] and ABCB5+ melanoma cells generate both ABCB5+ and ABCB5− progeny [262]. The direct implication of MITF in this phenotypic transition has also been demonstrated [85].

The existence of initiating cells in melanoma may explain the lack of objective responses to cancer treatment and the risk of recurrence observed in most cases. The standard treatment for melanoma is based on chemotherapy with alkylating agents (Decitene, Temodal, and Muphoran) targeting proliferative cells. It is therefore not surprising that these drugs are poorly efficient on the slow-growing melanoma-initiating cells. In addition, melanoma cells are able to reduce the intracellular drug accumulation by increasing the level pumps efflux, known as ABC transporters. ABCB5, presenting an increased expression during the malignant transformation [266], has been involved in the resistance of melanoma cells to doxorubicin [267]. Inhibition of ABCB5 dramatically improved intracellular drug accumulation and reduced the resistance of melanoma cells to doxorubicin [267].

The melanoma-initiating cells are able to escape the immune system. These cells are able to mask their antigenic identity inhibiting or not expressing such antigen MART-1 (melanoma antigen reconnu by T cells-1) recognized by T lymphocytes [268] or by inhibiting expression of the major histocompatibility complex type 1 (CMH-1) present antigenic peptides to lymphocytes [268]. For example, ABCB5 positive cells show no MHC-1 or MART-1. Interestingly, MITF regulates the expression of these proteins [30, 269], reinforcing our observations that cells expressing low levels of MITF correspond to tumor-initiating cells.

During these last few years, differences in the percentage of melanoma initiating cells (MIC) per tumor and the lack of consistent and reproducible markers of choice for MIC isolation from one study to another have engendered a stirring debate. Therefore, it is of paramount importance to identify appropriate and universal surface marker(s) to isolate and characterize the MICs. Biological characterization of the different cells within a tumor will help researchers to put together a complete portrait of the tumors and will contribute to design more efficient antimelanoma therapy [270].
